# Workplace Ergonomic Adjustments and Supportive Policies During the Pregnancy of a Paint Sprayer: A Case Report

**DOI:** 10.7759/cureus.76492

**Published:** 2024-12-27

**Authors:** Joshua A Jogie

**Affiliations:** 1 Faculty of Medical Sciences, The University of the West Indies, St. Augustine, TTO

**Keywords:** ergonomics, maternal health, occupational health, pregnancy, workplace adjustments

## Abstract

A 29-year-old paint sprayer presented with mild lower back pain and fatigue related to prolonged standing and repetitive tasks in the first few weeks of pregnancy. An initial assessment included a review of her workstation, evaluation of posture, and standard prenatal tests such as routine blood work and ultrasound. These investigations showed normal fetal development and no complications. The employer modified her work environment, providing ergonomic seating, and reducing prolonged standing. She received counseling on safe physical activity and balanced nutrition to help maintain overall health. Her pregnancy progressed without problems, and she continued to work comfortably. This case shows that simple ergonomic changes and supportive policies can help expectant employees remain productive and well.

## Introduction

Pregnancy introduces changes in posture, center of gravity, and joint laxity that can increase strain on the musculoskeletal system [1]. Women working in environments where repetitive physical exertion is common may be more likely to experience such discomfort [2]. Lower back soreness and fatigue are among the most common complaints among pregnant workers performing physically demanding jobs.

Occupational health considerations for pregnant workers have gained increasing attention in recent years. Many researchers and professionals emphasize that employers should consider the unique needs of pregnant employees and create safer, more ergonomic workplaces to maintain productivity and reduce health risks [3]. Fetal health and maternal comfort are both relevant factors when assessing work environments. If certain tasks strain a pregnant worker’s body, adjustments may be needed to prevent complications. Various organizations, including the American College of Obstetricians and Gynecologists (ACOG), have produced guidelines that encourage employers to consider job modifications for pregnant workers [4]. These guidelines do not suggest that pregnant individuals must cease working, but they highlight that minor workplace changes can help maintain maternal comfort, safety, and productivity.

Ergonomic interventions, such as the introduction of adjustable seating, reducing the duration of prolonged standing, and altering work tasks, can address some of the musculoskeletal challenges that pregnant workers face [5]. Adjusting a workstation or changing a worker’s responsibilities does not always require complicated or expensive measures. Simple changes can help reduce strain on the lower back, shoulders, and arms. Occupational exposure to chemicals is another concern for pregnant workers, especially those working with paints or solvents. Employers and clinicians must consider both chemical and physical risks [6]. Adequate ventilation, the use of personal protective equipment, and compliance with safety regulations help ensure that exposures remain within safe limits. Regular prenatal checkups, including routine blood tests and ultrasound examinations, serve as standard practices to monitor fetal growth and development [1]. Fatigue is another common complaint among pregnant workers. Changing hormonal levels, altered metabolism, and increased energy demands can make sustaining long work hours challenging [7]. Employers who understand these physiologic changes may choose to reduce task intensity or allow flexible working hours.

Employers who adopt a supportive and family-focused approach often see improved worker satisfaction and retention. A stable, engaged workforce may have less turnover, fewer workplace injuries, and improved morale. Safe physical activity during pregnancy is widely encouraged. Regular exercise supports cardiovascular health, reduces stress, and can alleviate common pregnancy discomforts [8]. Proper nutrition ensures that both mother and fetus receive essential nutrients. Proactive policies benefit both employers and employees. When companies implement guidelines that support expectant mothers, these workers may feel more secure, valued, and willing to stay in their roles. Strong policies reduce the risk of pregnancy-related injury or sick leave, which can save an employer time and resources while supporting the health of the workforce. A comprehensive approach includes not only ergonomic changes but also regular communication and consultations. Employers, occupational health professionals, and obstetric care providers should maintain an open dialogue. This helps identify issues early and respond promptly. For instance, if new discomfort arises or job tasks change, adjustments can be made quickly. Regular check-ins help ensure that the worker’s comfort and safety remain priorities.

Studies show that occupational activities, if well managed, do not necessarily harm pregnancy outcomes [3]. Many pregnant individuals can continue working in physically demanding jobs if proper precautions and modifications are implemented. Identifying risk factors involves a thorough assessment of job tasks, posture, environmental conditions, and exposure to harmful substances. Employers must recognize that not all workers share the same physical capabilities or vulnerabilities. Individual assessments are essential. Sometimes small adjustments like ergonomic seating, shorter periods of standing, and slight modifications to tasks can prove sufficient.

The medical literature suggests that many maternal concerns related to occupational activities stem from repetitive strain, excessive lifting, or continuous standing without breaks [5]. Reducing these stressors can prevent complications like severe back pain, sciatic nerve compression, or even preterm labor in high-risk cases. Proper intervention can prevent such escalation, leading to a more positive experience for the worker and the employer.

The concept of a family-focused corporate culture is supported by the notion that employees who feel cared for by their company show greater loyalty and engagement [2]. While this may appear to be a non-medical consideration, a supportive atmosphere indirectly influences health outcomes. Pregnant employees who believe their employer values their well-being may be more likely to report symptoms early. Early reporting allows for timely interventions. This can prevent workplace injuries, reduce absences, and maintain productivity.

Continuing ergonomic improvements and ongoing counseling throughout pregnancy can help the worker adapt to changing physical demands as the pregnancy progresses. For example, as the abdomen enlarges and the center of gravity shifts, tasks that were comfortable early in pregnancy may become more challenging later on [4]. Adjusting work assignments over time and reinforcing proper body mechanics ensures that the worker remains safe. Ongoing communication between the worker, employer, and healthcare providers helps monitor for new symptoms or changes in fetal well-being. Simple prenatal evaluations, including blood pressure checks, fetal heart rate monitoring, blood tests, and ultrasound examinations, confirm that the fetus is developing normally [1]. These investigations help reassure the worker and the employer that the work environment remains safe.

The current literature on occupational health in pregnancy suggests that systematic interventions, even simple ones, can improve maternal comfort and possibly reduce the likelihood of adverse outcomes [6,7]. By considering ergonomic factors and offering personalized adjustments, employers meet both legal and ethical responsibilities. They also support maternal and fetal health. This encourages a culture that values workers as more than just labor, recognizing their changing needs over time. In the long run, well-implemented policies may contribute to better health outcomes, stable employment, and overall job satisfaction.

This report describes the case of a 29-year-old woman working as a paint sprayer who had a physically demanding job that required prolonged standing and repetitive arm movements and experienced mild lower back soreness and fatigue during the early stages of her pregnancy. The changes made by the employer and this case demonstrates the potential benefits of a thoughtful approach to maternal occupational health. The paint sprayer maintained her job duties, stayed active, and felt supported, all while carrying a healthy pregnancy to term. The company’s response shows that straightforward changes can ensure pregnant workers remain part of the active workforce without increasing risks. Policies that encourage such supportive measures could serve as models for other industries.

## Case presentation

A 29-year-old woman working as a paint sprayer in a manufacturing setting learned she was pregnant following a home pregnancy test and subsequent confirmation by her primary care provider. At the time of discovery, she reported feeling generally well, but within a few weeks, she noticed mild lower back soreness and fatigue that seemed related to her extended shifts. Her typical workday involved prolonged standing, repetitive arm movements, and periods of bending to reach equipment and materials. Though her back soreness was not severe, it was persistent enough to make her worry about whether continuing her regular duties might affect her health or the health of the developing fetus. She had never been pregnant before, and she did not know what changes might occur in her body over the coming months. She understood that her musculoskeletal system would experience increased strain due to changes in posture and weight distribution, but she also knew that many women continue working throughout pregnancy. She decided to report her concerns to the on-site occupational health provider and her supervisor.

Initial evaluation by the company’s occupational health team included a detailed assessment of her work tasks, posture, and movement patterns. The provider observed her workstation, noting the height of the equipment she used and the positioning of the paint sprayer components. The provider also measured the time she spent standing without breaks, how frequently she reached overhead, and how far she had to bend. The occupational health provider performed a basic musculoskeletal exam, checking for tenderness along the lumbar area, evaluating her posture, and asking about the duration and intensity of her soreness. Her symptoms appeared mild and localized, and she denied any sharp pain or radiation into her legs. She had no prior history of chronic back pain or injuries, and her discomfort seemed closely tied to the physical demands of her job.

The patient's prenatal care also began around this time. She saw an obstetrician who performed standard first-trimester evaluations. Routine laboratory tests included a complete blood count, blood typing, Rh status, rubella titer, and screening for syphilis, hepatitis B, and HIV (Table 1). These tests followed standard prenatal screening protocols and did not show any abnormalities. A urinalysis was done to check for infection or proteinuria; it was normal (Table 1). An initial obstetric ultrasound confirmed a single intrauterine pregnancy with expected fetal development for gestational age. The fetal heartbeat was present, and measurements indicated a healthy embryo with no obvious structural issues. The obstetrician also recorded her blood pressure and weight, both of which fell within normal ranges for early pregnancy. This early confirmation of a healthy pregnancy reassured her that her mild musculoskeletal symptoms were likely manageable with basic interventions.

**Table 1:** First Trimester Laboratory Investigations

Parameters	Patient Values	Reference Range
Hemoglobin (g/dL)	13.2	11.0–14.0 (pregnancy)
Hematocrit (%)	38	33–40 (pregnancy)
White Blood Cell Count (/µL)	7,500	4,500–11,000
Platelets (/µL)	220,000	150,000–400,000
Blood Group (ABO, Rh)	O positive	N/A
Rubella IgG	Positive (immune)	Positive = immune
HIV	Negative	Negative
Hepatitis B Surface Ag	Negative	Negative
RPR (Syphilis)	Negative	Negative
Urinalysis (Protein)	Negative	Negative
Urinalysis (Glucose)	Negative	Negative

With the occupational health findings and her prenatal status in hand, the company decided to adjust her work environment. They recognized that long periods of standing could contribute to muscle fatigue and back discomfort, so they provided an adjustable ergonomic chair with proper lumbar support. This allowed her to sit for portions of her shift rather than remain standing the entire time. They also arranged for rotating job tasks that involved less bending and fewer repetitive overhead movements. When possible, she performed tasks at a comfortable height so that she did not need to stoop or stretch repeatedly. The goal was to maintain productivity while reducing strain on her back. The occupational health provider suggested short, periodic breaks for stretching and walking at a comfortable pace to keep her muscles from tightening and to improve circulation.

In addition to the ergonomic changes, the company offered her counseling from a staff health educator who specialized in maternal health in the workplace. This counseling session focused on practical advice for maintaining comfort and well-being. The educator explained that mild back soreness is common in early pregnancy as the body adapts to hormonal changes and shifting weight distribution. She encouraged the worker to engage in safe physical activities that promote strength and flexibility. Simple stretching exercises, guided by her obstetrician’s general recommendations, could help reduce tension in her back and hips. Gentle walks during breaks, if feasible, could improve circulation and energy levels. The counselor also emphasized balanced nutrition, suggesting frequent, small meals to maintain stable energy levels and recommending nutrient-dense foods rich in protein, complex carbohydrates, vitamins, and minerals. Adequate hydration was stressed, as fatigue can worsen if fluid intake is insufficient.

As her pregnancy progressed into the second trimester, she continued her regular prenatal visits. At each visit, the obstetrician measured her blood pressure, monitored her weight gain, and listened to the fetal heartbeat using a Doppler device. These routine checks reassured both the patient and the healthcare providers that the pregnancy was developing normally. Additional prenatal tests included a second-trimester ultrasound to assess fetal anatomy more thoroughly. This scan confirmed normal fetal growth, with no abnormalities detected. Blood tests at this stage, like screening for gestational diabetes around 24-28 weeks (commonly using an oral glucose tolerance test), ensured that no metabolic complications arose (Table 2). She showed no signs of gestational diabetes or hypertension. The obstetrician also inquired about her comfort at work and whether any adjustments were still needed.

**Table 2:** Second Trimester Laboratory Investigations

Parameters	Patient Values	Reference Range
Hemoglobin (g/dL)	12.0	10.5–14.0 (pregnancy)
Hematocrit (%)	35	32–40 (pregnancy)
White Blood Cell Count (/µL)	8,500	4,500–11,000
Platelets (/µL)	210,000	150,000–400,000
1-hour Glucose Challenge (mg/dL)	120	<140
Urinalysis (Protein)	Negative	Negative
Urinalysis (Glucose)	Negative	Negative

With the ergonomic interventions in place, the paint sprayer reported a noticeable reduction in back soreness. She felt more comfortable being able to alternate between sitting and standing. The reduced time spent bending or holding uncomfortable positions relieved pressure on her lower back. She also appreciated that the company encouraged regular breaks, allowing her a short period to sit quietly, stretch, or simply change her posture. These strategies prevented muscle fatigue from building up. Fatigue, while still present at times, became more manageable. Knowing that these modifications were approved and encouraged made her feel supported by her employer and improved her sense of well-being at work.

Throughout the pregnancy, no complications arose. She had routine follow-ups with the occupational health team, who continued to evaluate her workstation and tasks. When necessary, they made small changes to accommodate her growing abdomen and shifting center of gravity. For instance, as her pregnancy advanced, they made sure that items she needed to access remained at waist level, reducing the need for lifting or bending. She wore comfortable, supportive footwear to help maintain proper alignment and reduce stress on her back and legs. If a certain painting task required sustained reaching, a colleague could assist or they would rearrange the workflow so she could avoid awkward positions.

The prenatal visits continued at regular intervals. The obstetrician would ask about fetal movements, overall energy levels, and any new symptoms. Measurements of fundal height, fetal heart rate, and maternal vital signs remained normal. Urine screenings for protein and glucose stayed within normal limits (Table 3). By the third trimester, she was accustomed to her modified work routine and reported feeling capable of performing her duties without significant discomfort.

**Table 3:** Third Trimester Laboratory Investigations

Parameters	Patient Values	Reference Range
Hemoglobin (g/dL)	11.8	10.5–14.0 (pregnancy)
Hematocrit (%)	34	32–40 (pregnancy)
White Blood Cell Count (/µL)	9,000	4,500–11,000
Platelets (/µL)	200,000	150,000–400,000
Urinalysis (Protein)	Negative	Negative
Urinalysis (Glucose)	Negative	Negative

As the pregnancy approached term, she maintained a positive outlook and was able to keep working. By the time she reached the later weeks of pregnancy, she was confident that she could continue working safely until her planned maternity leave. She understood that if circumstances changed, the company and healthcare providers would reassess and make further modifications as needed. The absence of complications and stable maternal and fetal well-being demonstrated that the approach taken was effective.

## Discussion

This case highlights how a pregnant worker with mild musculoskeletal discomfort and fatigue can benefit from simple yet effective ergonomic modifications and supportive workplace policies. The observations in this case add to existing knowledge on how tailored adjustments can help expectant employees remain comfortable and productive throughout pregnancy. Many studies have considered the impact of work-related physical demands on maternal health and fetal outcomes. In this case, while the worker experienced discomfort, it was effectively managed by combining ergonomic changes with regular monitoring, underscoring the importance of proactive interventions. However, when employers recognize these risks and make appropriate changes, pregnant workers can often continue their duties without complications.

The mild lower back soreness the patient reported aligns with common musculoskeletal challenges that occur during pregnancy. Changes in the maternal body involve shifts in posture, increased joint laxity, and a growing uterus that alters the center of gravity. These changes can exacerbate strain on the lower back and contribute to fatigue. Prolonged standing and repetitive tasks, as seen in this paint sprayer’s job, can aggravate these discomforts. Research suggests that physically demanding work, particularly when it involves awkward postures or limited rest, can elevate the risk of musculoskeletal issues during pregnancy [9]. Nonetheless, not all physically demanding work leads to complications. It often depends on the presence of mitigating factors, such as adjustable equipment and break schedules, as well as the intensity and duration of the tasks performed.

In this case, ergonomic interventions made a difference. Adjusting her tasks to reduce extended standing, providing proper seating, and ensuring more comfortable positions reduced the strain on her back. Studies show that such interventions can help decrease musculoskeletal complaints by promoting better posture and reducing static load [10]. Improving the ergonomic environment does not require expensive solutions. Sometimes, something as simple as providing a chair with lumbar support or rearranging the workstation to avoid bending and overhead reaching can offer substantial relief. Evidence suggests that such adjustments can prevent minor discomforts from escalating into more severe problems [11]. By addressing these issues early, employers help maintain the worker’s health, prevent lost work time, and reduce potential costs associated with medical care or absenteeism.

Safe physical activity during pregnancy is generally encouraged, and a balanced approach can improve maternal health. Maintaining moderate physical activities, as advised in this case by counseling sessions, supports cardiovascular function, reduces stress, and can help control weight gain. Some studies associate excessive physical exertion at work with an increased risk of preterm birth and low birth weight, but these risks may be mitigated by managing the intensity and duration of activities [12]. Short breaks, task rotations, and attention to posture can allow pregnant workers to stay active without exposing themselves or their unborn children to unnecessary risks. The patient in the current case followed the advice on balanced nutrition and maintained safe physical activity, and her pregnancy proceeded without complications. This outcome reinforces findings that physical activities, when appropriately managed, do not inherently harm pregnancy outcomes [13].

From a broader perspective, the supportive company policies in this scenario underscore the importance of workplace culture. When employers adopt family-focused policies and provide resources for expectant workers, they foster a sense of trust and well-being. This can improve workforce engagement and morale. Employees who feel supported are more likely to report early symptoms of discomfort. Early reporting leads to timely interventions that can prevent more severe complications down the line. By making simple changes, the employer created an environment that valued the pregnant worker’s health and that of her developing child. This approach aligns with research showing that employers who recognize reproductive health issues and implement preventive strategies can reduce the likelihood of adverse maternal and fetal outcomes [14].

Supportive policies also help reduce stress, another factor that can affect pregnancy health. Excessive workplace stress may contribute to fatigue and other maternal complaints. A stable and supportive environment can lower stress levels, potentially improving sleep quality and energy management. When pregnant workers perceive that their concerns will be addressed, they may experience less anxiety and feel more confident continuing their duties. Evidence supports the notion that work conditions that account for pregnancy-related changes can help ensure healthy progress of pregnancy while maintaining the mother’s ability to work [15]. This case fits that pattern. The paint sprayer felt supported, her symptoms improved, and she remained engaged and comfortable at work.

Routine prenatal care and regular investigations were essential to confirm fetal well-being. Standard blood tests, blood pressure checks, and ultrasound examinations verified that the fetus was developing normally, and no complications arose. These routine investigations help differentiate common pregnancy discomforts from serious conditions. Frequent prenatal check-ups can identify potential issues early, allowing timely adjustments or medical interventions if needed. The absence of complications in this case, coupled with ongoing monitoring, supports the idea that pregnant workers can maintain employment in a well-managed setting without harm to fetal development [16]. Employers who encourage regular prenatal care and provide scheduling flexibility contribute to maintaining a healthy pregnancy.

In many industries, pregnant employees fear that reporting discomforts could lead to reassignment to less desirable positions or even job loss. This apprehension may cause them to continue enduring strain, which can worsen their symptoms. Employers who proactively encourage open communication create a safer environment. Early intervention benefits everyone. The worker maintains health and productivity, while the employer avoids the costs associated with injury, absenteeism, or turnover. Over time, this builds a positive culture that retains skilled employees and signals to potential hires that the company invests in its workforce.

Another point is that pregnancy is not a one-size-fits-all condition. Different women have different tolerance levels, and the physical demands that might be manageable for one pregnant worker might be challenging for another. Individualized assessments help ensure that solutions are tailored. In this case, the paint sprayer presented mild symptoms that were easily addressed. In other situations, a pregnant worker might require more extensive job modifications or even temporary reassignment if risks cannot be mitigated. The key is flexibility and willingness to adapt to the worker’s changing needs, which may shift as the pregnancy progresses. Midway through pregnancy, increased abdominal size and altered posture may necessitate further tweaks to the workstation. The goal is to keep tasks manageable and safe at all stages.

When workplace interventions align with medical guidance, expectant employees receive consistent messages about their health. It reassures them that continuing work is safe and, with proper precautions, may even be beneficial. Moderate physical activity, after all, can help maintain fitness and may reduce some common pregnancy complaints like fluid retention and mild edema. The company’s counseling on balanced nutrition and safe exercises complemented the ergonomic changes. Good nutrition supports maternal health and fetal development, ensuring that the worker maintains sufficient energy levels to perform her tasks.

Work modifications, maternal counseling, and ongoing prenatal care work together to ensure safety and comfort [10-12]. Regular monitoring keeps everyone informed about the pregnancy’s status and any emerging risks. No single measure guarantees a complication-free pregnancy, but a combination of careful assessments, responsive interventions, and supportive policies can come close. This multi-faceted approach aligns with known best practices for occupational health in pregnancy [13,14].

In addition, addressing pregnancy-related issues at work can improve long-term health and economic outcomes. Workers who remain active and employed during pregnancy might experience fewer financial strains and maintain a stable environment for their growing families. Employers who invest in supportive measures may see reduced turnover and enhanced worker loyalty. This can translate into cost savings and a more stable workforce over time. Such policies can form part of a broader strategy for workforce well-being, encompassing not just pregnant workers but all employees with different needs. Moderate musculoskeletal discomfort in pregnancy need not lead to absence from work if the environment can be adapted. The literature and this patient’s experience both support the assertion that small adjustments can yield significant benefits. Ergonomic improvements, flexible scheduling, and nutritional and physical activity counseling help maintain maternal health and productivity. Preventive steps are often more effective than reactive ones, so implementing these measures early in pregnancy is wise.

Finally, the stable course of this patient’s pregnancy and her comfort at work suggest that family-focused and proactive policies align with the interests of both employer and employee. A careful blend of medical guidance, occupational health assessments, and responsive management promotes favorable outcomes. It also reinforces the idea that pregnancy is a natural phase of life that can be managed safely in the workplace. This reflects broader trends in occupational health research, which emphasize adaptation, prevention, and open communication to support pregnant workers [15,16].

## Conclusions

This case shows that simple ergonomic changes and supportive policies can help pregnant workers remain comfortable, healthy, and productive. Adjusting tasks to reduce prolonged standing and providing ergonomic seating eased mild lower back soreness and fatigue. Incorporating interdisciplinary collaboration, where occupational health experts work alongside obstetricians, ensures a tailored and evidence-based approach to pregnancy care in the workplace. Counseling reinforced safe physical activity, balanced nutrition, and open communication. Taken together, these measures supported the worker through her pregnancy, allowing her to continue working without complications. This experience suggests that thoughtful workplace adjustments and regular monitoring can benefit both pregnant employees and their employers.
